# Mental illness and lost income among adult South Africans

**DOI:** 10.1007/s00127-012-0587-5

**Published:** 2012-09-25

**Authors:** Crick Lund, Landon Myer, Dan J. Stein, David R. Williams, Alan J. Flisher

**Affiliations:** 1 Alan J Flisher Centre for Public Mental Health, Department of Psychiatry and Mental Health, University of Cape Town, 46 Sawkins Road, Rondebosch, Cape Town, 7700 South Africa; 2 School of Public Health and Family Medicine, University of Cape Town, Cape Town, South Africa; 3 Harvard University School of Public Health, Boston, MA USA; 4 Department of Psychiatry and Mental Health, University of Cape Town, 46 Sawkins Road, Rondebosch, Cape Town, 7700 South Africa; 5 Division of Child and Adolescent Psychiatry, Department of Psychiatry and Mental Health, University of Cape Town, Cape Town, South Africa

**Keywords:** Income, Mental disorder, South Africa, Economics, Health policy

## Abstract

**Purpose:**

Little is known regarding the links between mental disorder and lost income in low- and middle-income countries. The purpose of this study was to investigate the association between mental disorder and lost income in the first nationally representative psychiatric epidemiology survey in South Africa.

**Methods:**

A probability sample of South African adults was administered the World Health Organization Composite International Diagnostic Interview schedule to assess the presence of mental disorders as defined in the Diagnostic and Statistical Manual of Mental Disorders, version IV.

**Results:**

The presence of severe depression or anxiety disorders was associated with a significant reduction in earnings in the previous 12 months among both employed and unemployed South African adults (*p* = 0.0043). In simulations of costs to individuals, the mean estimated lost income associated with severe depression and anxiety disorders was $4,798 per adult per year, after adjustment for age, gender, substance abuse, education, marital status, and household size. Projections of total annual cost to South Africans living with these disorders in lost earnings, extrapolated from the sample, were $3.6 billion. These data indicate either that mental illness has a major economic impact, through the effect of disability and stigma on earnings, or that people in lower income groups are at increased risk of mental illness. The indirect costs of severe depression and anxiety disorders stand in stark contrast with the direct costs of treatment in South Africa, as illustrated by annual government spending on mental health services, amounting to an estimated $59 million for adults.

**Conclusions:**

The findings of this study support the economic argument for investing in mental health care as a means of mitigating indirect costs of mental illness.

## Introduction

The cost of mental illness is complex and difficult to measure. Yet, it is an important indicator of the economic burden of mental illness to a society. Traditionally, cost of mental illness studies has been divided into direct and indirect costs [1]. Indirect costs tend to outweigh direct costs in most studies [1]. Among indirect costs, the cost of lost income due to mental illness is an important element.

According to Amartya Sen, economic development needs to take into account not only the basket of goods (such as income and assets) that a person holds but also the relevant personal characteristics that govern how the primary goods are converted into the person’s ability to promote her or his ends [2]. For example, a person who is disabled may have a larger basket of primary goods but be less able to lead a normal life, or pursue her or his objectives, than another person with a smaller basket of primary goods. Mental health enters this framework as a set of “functionings” that enable the various things a person may value doing or being: “a person’s “capability” refers to the alternative combinations of functionings that are feasible for her to achieve” [2] (p.75). Taken together, mental health and income provide a basis for optimizing choice in the consumption of goods and services, as well as pursuit of valued life choices.

Mental disorders lead to lost income through the disabilities and stigma with which they are associated [3] and negatively influence a person’s ability to convert available income into capability—both key elements in the social selection or social drift pathway in the cycle of poverty and mental illness [4]. Conversely, low income increases the risk for mental disorders through increased risk of adverse life events and reduced access to resources that can buffer the effects of those life events—contributing to the social causation of mental illness [5]. A recent study from the United Kingdom has provided evidence of both social causation and social selection in the long-term predictors of adult depression and anxiety disorders [6].

Most studies showing an association between mental disorders and reduced income have been conducted in high-income countries [1, 7]. In low- and middle-income countries (LMICs), little is known regarding the links between mental disorder and reduced income, despite evidence of a substantial burden of mental illness [8, 9] and severely under-resourced mental health care systems [10, 11]. Studies have been conducted of indirect costs of mental illness in Taiwan [12] and Kenya [13], but both of these have adopted a human capital approach, which focuses on estimates of lost productivity, rather than reported lost income. Losses in income or paid production are particularly threatening (in terms of impoverishment) in low- and middle-income settings where compensation mechanisms such as welfare or disability benefits for mental illness are unavailable or limited.

Data regarding mental illness and lost income can provide estimates of some of the indirect economic costs of mental illness to these societies and strengthen the economic argument for investment in mental health care as a means of mitigating these costs and promoting more broad-based economic development. This is particularly pertinent in the light of emerging evidence from LMICs that providing mental health care can reduce disability and yield economic benefits to individuals and their households [14]. This study sets out to report on the association between mental disorder and lost income in the first nationally representative psychiatric epidemiology study in South Africa.

## Methods

The South African Stress and Health (SASH) Study is a national survey of mental health conducted between January 2002 and June 2004. The study rationale, methods, and ethics approval have been described in detail previously [15, 16]. A probability sample of 4,351 South African adults (age ≥ 18 years) living in both households and hostel quarters was selected using a three-stage design. The response rate was 85.5 %, and individuals of all major racial and ethnic groups were included. The sample was weighted to ensure representation of each of the main ethnic groups [15]. All analyses employed person-level weights to account for the complex survey design, with adjustments for sample selection, non-response, and post-stratification factors as previously described. Calculations for estimation and inference were based on the Taylor series linearization method.

The World Health Organization (WHO) Composite International Diagnostic Interview Version 3.0 (CIDI) [17] was used to assess the presence of mental disorders as defined in the Diagnostic and Statistical Manual of Mental Disorders, version IV (DSM-IV) [18]. The CIDI is a fully structured diagnostic interview that is lay administered and can generate diagnoses according to both the WHO International Classification of Diseases (ICD-10) and the DSM-IV diagnostic systems. The translation of the English version of CIDI into the six other South African languages used in the SASH study was carried out according to WHO recommendations of iterative back-translation conducted by panels of bilingual and multilingual experts. Discrepancies found in the back-translation were resolved by consensus of an expert panel.

The mental disorders assessed in the SASH study were anxiety disorders [panic disorder, agoraphobia, social phobia, generalized anxiety disorder (GAD), post-traumatic stress disorder (PTSD)], mood disorders (major depressive disorder, dysthymia), substance disorders (alcohol abuse, alcohol dependence, drug abuse, drug dependence), and intermittent explosive disorder. DSM-IV organic exclusion rules and diagnostic hierarchy rules were applied to all diagnoses, except in the case of substance use disorders where abuse was defined with or without dependence. Disorders such as schizophrenia and bipolar mood disorder were not included in the SASH survey, as these disorders would require a clinical assessment, and the available resources for the study were only able to support lay administered instruments such as the CIDI.

In the analysis of lost income, only severe depression and anxiety disorders were included. Respondents were included if they (a) satisfied the criteria for any of the following disorders in the previous 12 months: major depressive episode, agoraphobia, PTSD, GAD, social phobia, specific phobia, intermittent explosive disorder, adult separation anxiety, and dysthymia and (b) either attempted suicide in the past 12 months or had a high level of impairment in the social, family, occupational, or study domains.

All respondents were asked to report their personal earnings in the past 12 months, before taxes. Respondents were instructed to count only wages and other stipends from employment, not pensions, investments, or other financial assistances or income (such as grants).

Analysis followed the same approach as that of the National Comorbidity Survey Replication in the United States of America [7]. Multiple regression analysis was conducted of 12-month personal earnings on those with DSM-IV mental disorders, controlling for age, gender, substance abuse, education, marital status, and household size. Projections of total annual cost to South Africa in lost earnings were extrapolated from the sample by multiplying the lost earnings per individual with severe depression or anxiety disorders by the prevalence of these disorders and the total population. We used generalized linear regression models to examine the associations between severe depression and anxiety disorders (as the independent variable) with different measures of lost income (as dependent variables). First, we examined 12-month earnings as a continuous variable, modeled with a logarithmic link function and normal error distribution; the resulting coefficients can be interpreted as the mean difference in 12-month income, comparing individuals with and without severe depression and anxiety disorders. Normality was tested using standard methods (for example Kolmogorov–Smirnov test). We ran separate models for both employed and unemployed individuals and employed individuals only. In addition, we examined any income in the preceding 12 months as a binary-dependent variable, modeled with a logit function; the resulting odds ratios can be interpreted as the relative odds of reporting any income in the preceding 12 months for individuals with and without severe depression and anxiety disorders. All models were adjusted for participant sex, age, alcohol dependence (12 months), alcohol abuse without dependence (12 months), drug dependence (12 months), drug abuse without dependence (12 months), alcohol dependence (lifetime), alcohol abuse without dependence (lifetime), drug dependence (lifetime), and drug abuse without dependence (lifetime).

All recruitment, consent, and field procedures were approved by the Human Subjects Committees of the University of Michigan, Harvard Medical School, and by a single project assurance of compliance from the Medical University of South Africa that was approved by the National Institute of Mental Health. The research conforms to the principles embodied in the Declaration of Helsinki.

## Results

In the sample as a whole, major depressive disorder, agoraphobia, and alcohol abuse were the most prevalent DSM-IV disorders. Anxiety disorders were the most prevalent group of disorders, followed by substance use disorders. The number of people from whom data on lost earnings were collected was 4,074, comprising 2,436 women and 1,638 men. The prevalence estimates were 3.3 % for severe 12-month depression and anxiety disorders, 10.1 % for all other (non-severe) 12-month disorders, and 10.1 % for other lifetime disorders (Table 1). There were significant gender differences in the prevalence of these disorders (*p* < 0.05). There were also significant gender differences in all substance use disorders (*p* < 0.05), with the exception of 12-month drug dependence. Only 37.7 % of the sample reported any earnings in the previous 12 months. Significantly more men had any earnings in the previous 12 months than women (*p* < 0.05).

**Table 1:** Clinical and sociodemographic characteristics

	Total (*N* = 4,074) % (SE)		Male (*N* = 1,638) % (SE)		Female (*N* = 2,436) % (SE)		Male vs. female χ^2^-test
I. Mental disorders							9.16*
12-month severe depression/anxiety**	3.25	0.28	2.19	0.39	4.17	0.49	
Other 12-month disorders	10.12	0.68	7.95	0.91	12.00	0.86	
Other lifetime disorders	10.10	0.64	8.97	0.81	11.09	0.85	
II. Outcomes							27.99*
Any 12-month earnings	37.68	1.26	42.35	1.54	33.60	1.46	
Earning categories							1.52
Low earnings^a^	45.85	2.25	43.53	3.01	48.39	2.40	
Low-average earnings^a^	28.13	1.47	28.92	2.12	27.26	1.82	
High-average earnings^a^	26.02	1.95	27.54	2.36	24.35	2.13	
III. Socio-demographic controls							
Sex							
Male	46.55	0.98					
Female	53.45	0.98					
Age							3.52
18–24	24.73	0.85	26.42	1.19	23.26	1.14	
25–39	39.41	0.89	39.96	1.50	38.94	0.96	
40–54	26.51	0.92	25.73	1.36	27.18	0.95	
55–64	9.35	0.57	7.89	0.76	10.62	0.76	

* Significant sex difference at the 0.05 level, two-sided test

** Severe depression and anxiety disorders, as defined in “Methods” section

^a^Earnings were defined as follows low earnings (<4,500.00), low-average earnings (≥4,500.00 to <16,500.00), high-average earnings (≥16,500.00)

The presence of severe mental disorder was associated with a significant loss of income in the previous 12 months among South African adults (Table 2). In Model 1 (employed and unemployed), severe depression and anxiety disorders were associated with reduced income for the entire sample, including both employed and unemployed groups (*p* = 0.0043). In Model 2, severe depression and anxiety disorders were not associated with the presence of income (*p* = 0.99). In Model 3, severe depression and anxiety disorders were associated with reduced earnings among employed people only (*p* = 0.0063).

**Table 2:** Generalized linear model estimates of the association between mental disorders and 12-month earnings

	Coefficient estimate^a^	SE	Odds ratio	95 % CI		*p* value
				Lower	Upper	
Model I (employed and unemployed)						
Severe depression and anxiety disorders (in last 12 months)	−1.1857	0.3996				0.0043
Model II (income yes or no)						
Severe depression and anxiety disorders (in last 12 months)			1.0015	0.6292	1.5942	0.9948
Model III (employed only)						
Severe depression and anxiety disorders (in last 12 months)	−1.1235	0.3971				0.0063

Control variables include sex, age, alcohol dependence (12 months), alcohol abuse without dependence (12 months), drug dependence (12 months), drug abuse without dependence (12 months), alcohol dependence (lifetime), alcohol abuse without dependence (lifetime), drug dependence (lifetime), and drug abuse without dependence (lifetime)

Interaction variables include sex × severe depression/anxiety (12 months), sex × controls (listed above)

^a^Coefficient estimates in models I and III are based on generalized linear models with multiple linear regression using a logarithmic link function. The coefficient is the mean log difference in 12-month income. Odds ratio is presented for model II, which is based on a multiple logistic regression model

In simulations of costs to individuals, the mean estimated impact of severe depression and anxiety disorders was $4,798 per person, after adjustment for age, gender, substance abuse, education, marital status, and household size (Table 3). This impact was felt more acutely by women, among whom the mean estimated lost earnings associated with severe depression and anxiety disorders was $6,390 compared as $1,313 in men. Projections of total annual cost in lost earnings for South Africans with these disorders, extrapolated from the sample, are $3,626,666,995, assuming estimated lost earnings due to individuals with severe depression and anxiety disorders of $4,798, 12-month prevalence of 3.25 %, and the South African adult population (20–64 years) of 23,257,556 based on the 2001 South African census [19].

**Table 3:** Mean expected earnings in the absence of severe depression and anxiety disorders compared to observed earnings among respondents with 12-month DSM-IV/CIDI severe depression and anxiety disorders

	Total (*N* = 4,074)		Male (*N* = 1,638)		Female (*N* = 2,436)	
	Mean (US$)	SD	Mean(US$)	SD	Mean(US$)	SD
Respondents with severe depression/anxiety						
Mean observed earnings	4,949	7,930	9,065	10,224	3,067	4,196
Mean expected earnings	9,746	12,221	10,378	11,704	9,458	12,936
Mean estimated impact of illness	4,798	4,191	1,313	3,034	6,390	4,583
Total population						
Mean observed earnings	9,411	15,896	9,106	14,971	9,677	13,908
Mean expected earnings	9,567	16,170	9,135	15,005	9,943	14,536
Mean estimated impact of illness	156	732	29	452	267	926

Control variables include: sex, age, household size, alcohol dependence (12 months), alcohol abuse without dependence (12 months), drug dependence (12 months), drug abuse without dependence (12 months), alcohol dependence (lifetime), alcohol abuse without dependence (lifetime), drug dependence (lifetime), drug abuse without dependence (lifetime), marital status, and education

“Mean observed earnings” are the actual mean annual earnings of the respondents in US Dollars

“Mean expected earnings” are the mean annual earnings of respondents in the absence of severe depression and anxiety disorders

“Mean estimated impact of illness” is mean expected earnings–mean observed earnings

## Discussion

The findings of this study draw attention to the strong association between lost income and severe depression and anxiety disorders in South Africa. The total economic burden of mental illness is likely to be higher than that reported in this study, given the exclusion from this analysis of child and adolescent mental disorders and other severe chronic disorders such as schizophrenia and bipolar mood disorder. In separate analysis, child and adolescent mental disorders in the SASH survey were associated with reduced educational attainment and likely subsequent income [20]. Furthermore, the study does not take into account either direct economic costs of mental illness, such as those associated with treatment or other indirect costs such as transport to health facilities, lost income among caregivers, and disability grants. The hidden costs to carer and family members are particularly important and frequently difficult to assess [21].

The findings in relation to gender are particularly striking. Women are at increased risk for depression and anxiety disorders, showing a 12-month prevalence approximately twice that of men, in keeping with international studies [8]. In addition, the impact of these disorders on women’s income is much greater than it is for men. This difference may be partially attributed to increased prevalence but may also be due to the more disabling impact of these disorders in women, or the possibility that women may be earning income in more unstable informal settings, which are more vulnerable to losses of income associated with illness.

The findings support other research that indicates that mental illness, through its strong association with reduced earnings, appears to have a major socio-economic impact on LMICs. For example, previous studies from LMICs, although using a human capital approach to measuring indirect costs, indicate a loss of productivity due to depression of $1,053 million in Taiwan [12] and a loss of productivity due to 5,678 admissions to psychiatric hospitals of $2,569,719 in Kenya during the 1998/1999 financial year [13]. The findings are also supported by other data from South Africa [22, 23] and Brazil, Chile, Uganda, and Zimbabwe [24–27] that indicate an association between socio-economic adversity and increased risk for mental illness. However, the dearth of longitudinal studies in LMICs means that it is difficult to draw any clear conclusions regarding causality, from our current knowledge base. There is also a strong possibility that the associations in this study reflect the increasing vulnerability to depression and anxiety disorders among low-income groups in South Africa, described elsewhere as the social causation of mental illness [3].

The indirect costs of severe depression and anxiety disorders stand in stark contrast with the direct costs in South Africa, as illustrated by government spending on mental health services. In 2005, this was estimated to be 1, 5, and 8 % of total health expenditure in Northern Cape, North West, and Mpumalanga provinces, respectively [28]. Projected to the total South African adult population [19], this amounts to annual government expenditure of $59,325,103—a figure which is dwarfed by the estimated lost earnings of $3,626,666,995 among adults with severe depression and anxiety disorders alone. Although there are limitations to comparisons of this nature (treatment does not necessarily lead to full recovery of lost earnings), such data do provide support for the argument that it costs South African society more to not treat than to treat mental illness. This adds support to the economic argument for preventing mental illness [29] and scaling up mental health care and rehabilitation services [14, 30], as a means of mitigating the economic burden of these illnesses.

There are several limitations to this study which need to be noted. As acknowledged previously, there are limitations to the SASH study in the validity of the instrumentation and its applicability to all of the cultural groups in South Africa [16, 31]. This analysis of lost income has been conducted only for adults with severe depression and anxiety disorders and does not take into consideration the lost earnings among adults with mild to moderate mental illness, children, and adolescents with any disorders or adults with other severe mental illnesses such as schizophrenia and bipolar mood disorder. Furthermore, the study is cross-sectional, making it difficult to draw conclusions regarding the causal relationship between lost income and depression/anxiety disorders, a feature which is shared among many epidemiological studies that examine socio-economic correlates of mental ill-health in LMICs [32]. Thus, the association noted here may be attributable to either social causation (those with lower income are at greater risk of depression and anxiety disorders) or to social selection (those with depression and anxiety disorders are at greater risk of losing income through increased health expenditure, job loss and reduced productivity associated with the increased disability and stigma of their conditions). Finally, one cannot conclude that the lost income associated with mental illness is a broader societal loss. For example, the wages that are not paid to people with depression and anxiety disorders may be used to employ other previously unemployed people and may not lead to a decrease in consumption and the implication that there is a broader economic loss.

Future research needs to address some of these limitations by conducting longitudinal studies in LMICs, examining the effects of interventions on the relationship between socio-economic factors and mental illness [14], and exploring the impact of mild–moderate mental disorders on income and production.
